# Analysis of Salivary Mycobiome in a Cohort of Oral Squamous Cell Carcinoma Patients From Sudan Identifies Higher Salivary Carriage of *Malassezia* as an Independent and Favorable Predictor of Overall Survival

**DOI:** 10.3389/fcimb.2021.673465

**Published:** 2021-10-12

**Authors:** Nazar Mohamed, Jorunn Litlekalsøy, Israa Abdulrahman Ahmed, Einar Marius Hjellestad Martinsen, Jessica Furriol, Ruben Javier-Lopez, Mariam Elsheikh, Nuha Mohamed Gaafar, Luis Morgado, Sunil Mundra, Anne Christine Johannessen, Tarig Al-Hadi Osman, Elisabeth Sivy Nginamau, Ahmed Suleiman, Daniela Elena Costea

**Affiliations:** ^1^ Gade Laboratory for Pathology, Department of Clinical Medicine, and Center for Cancer Biomarkers CCBIO, University of Bergen, Bergen, Norway; ^2^ Department of Oral and Maxillofacial Surgery/Department of Basic Sciences, University of Khartoum, Khartoum, Sudan; ^3^ Department of Operative Dentistry, University of Science & Technology, Omdurman, Sudan; ^4^ Department of Clinical Science, University of Bergen, Bergen, Norway; ^5^ Department of Nephrology, Haukeland University Hospital, Bergen, Norway; ^6^ Department of Biological Sciences, The Faculty of Mathematics and Natural Sciences, University of Bergen, Bergen, Norway; ^7^ Department of Oral & Maxillofacial Surgery, Khartoum Dental Teaching Hospital, Khartoum, Sudan; ^8^ Section for Genetics and Evolutionary Biology (EvoGene), Department of Biosciences, The Faculty of Mathematics and Natural Sciences, University of Oslo, Oslo, Norway; ^9^ Department of Biology, College of Science, United Arab Emirates University, Al Ain, Abu Dhabi, United Arab Emirates; ^10^ Department of Pathology, Laboratory Clinic, Haukeland University Hospital, Bergen, Norway

**Keywords:** oral squamous cell carcinoma (OSCC), mycobiome, *toombak*, biomarker, overall survival (OS), *Malassezia*, *Candida*

## Abstract

**Background:**

Microbial dysbiosis and microbiome-induced inflammation have emerged as important factors in oral squamous cell carcinoma (OSCC) tumorigenesis during the last two decades. However, the “rare biosphere” of the oral microbiome, including fungi, has been sparsely investigated. This study aimed to characterize the salivary mycobiome in a prospective Sudanese cohort of OSCC patients and to explore patterns of diversities associated with overall survival (OS).

**Materials and Methods:**

Unstimulated saliva samples (*n* = 72) were collected from patients diagnosed with OSCC (*n* = 59) and from non-OSCC control volunteers (*n* = 13). DNA was extracted using a combined enzymatic–mechanical extraction protocol. The salivary mycobiome was assessed using a next-generation sequencing (NGS)-based methodology by amplifying the ITS2 region. The impact of the abundance of different fungal genera on the survival of OSCC patients was analyzed using Kaplan–Meier and Cox regression survival analyses (SPPS).

**Results:**

Sixteen genera were identified exclusively in the saliva of OSCC patients. *Candida*, *Malassezia*, *Saccharomyces*, *Aspergillus*, and *Cyberlindnera* were the most relatively abundant fungal genera in both groups and showed higher abundance in OSCC patients. Kaplan–Meier survival analysis showed higher salivary carriage of the *Candida* genus significantly associated with poor OS of OSCC patients (Breslow test: *p* = 0.043). In contrast, the higher salivary carriage of *Malassezia* showed a significant association with favorable OS in OSCC patients (Breslow test: *p* = 0.039). The Cox proportional hazards multiple regression model was applied to adjust the salivary carriage of both *Candida* and *Malassezia* according to age (*p* = 0.029) and identified the genus *Malassezia* as an independent predictor of OS (hazard ratio = 0.383, 95% CI = 0.16–0.93, *p* = 0.03).

**Conclusion:**

The fungal compositional patterns in saliva from OSCC patients were different from those of individuals without OSCC. The fungal genus *Malassezia* was identified as a putative prognostic biomarker and therapeutic target for OSCC.

## Introduction

The oral cavity is a habitat for a diverse and fluctuating collection of microorganisms (Aas et al., 2005; Nasidze et al., 2009; Yang et al., 2016). The oral microbiome, which includes, in addition to complex bacterial communities, oral fungi, viruses, and phages (Baker et al., 2017), is one of the most diverse microbial communities in the human body (Dewhirst et al., 2010; Huttenhower et al., 2012), and this is related to its multiple ecosystems (Arweiler et al., 2016). The oral microbiota represents a critical component of health and diseases (Jenkinson and Lamont, 2005; Avila et al., 2009), and balance is maintained by a continuous interplay with the host (Vasquez et al., 2018). Dysbiosis of the oral microbiome has been proposed as a marker, initiator, or modifier of oral diseases (Ghannoum et al., 2010; Hooks and O’Malley, 2017; Iliev and Leonardi, 2017; Rosier et al., 2018).

Recent advances in microbial detection techniques allowed the transition from culture-dependent studies of a single species to complex *in vitro* multispecies community detection and characterization studies (Baker et al., 2017). Large next-generation sequencing (NGS)-based projects, such as the Human Microbiome (Huttenhower et al., 2012), the Integrative Human Microbiome Project with a focus on the mechanisms of host–microbiome interactions (Proctor et al., 2019), and the Human Oral Microbiome Database (Proctor et al., 2019), give deeper insights into the human microbiome. Despite advances in the understanding of the microbiome, majority of the studies have focused on the bacterial part of the microbiome. Little is known about the fungal part of the human microbiome, recently defined as the mycobiome (Ghannoum et al., 2010; Cui et al., 2013; Chandra et al., 2016).

The few existing studies have revealed that the diversity of the oral mycobiota is lower when compared to that of the oral bacteriome (Iliev and Leonardi, 2017), and it is dominated by members of the phylum Ascomycota, mainly *Candida* spp., with *Candida albicans* as the dominant species. The other commonly identified fungi in the oral mycobiome are *Cladosporium*, *Aureobasidium*, *Saccharomycetales*, *Aspergillus*, *Fusarium*, *Cryptococcus*, and *Malassezia* (Ghannoum et al., 2010; Dupuy et al., 2014).

Evidence is accumulating on the role of fungi in neoplasia (Rindum et al., 1994; McCullough et al., 2002; Barrett et al., 2008; Hebbar et al., 2013; Berkovits et al., 2016; Zhu et al., 2017; Conche and Greten, 2018; Al-Hebshi et al., 2019; Aykut et al., 2019). Some earlier studies have suggested a possible role of *Candida* in the initiation of carcinogenesis (Field et al., 1989; Krogh, 1990). *Candida* may have a causal role in oral precancer and cancer, albeit an indirect one, implying that *Candida*, along with other cofactors, e.g., tobacco consumption, is involved in the initiation and promotion of carcinogenesis (Bakri et al., 2010; Sanjaya et al., 2011). Some *C. albicans* strains may contribute to oral carcinogenesis by producing endogenous nitrosamine (Krogh et al., 1987). An immune-mediated role in the acceleration of pancreatic ductal adenocarcinoma has also been suggested recently for another genus, namely, *Malassezia* (Aykut et al., 2019).

There are only sparse reports in the literature on the mycobiome in oral squamous cell carcinoma (OSCC). Perera et al. revealed a dysbiotic mycobiome characterized by lower species diversity and increased relative abundance of *C. albicans* in tissue biopsies of OSCC in a cohort of patients from Sri Lanka (Perera et al., 2017). Berkovits et al. (2016) used cultivation techniques coupled with matrix-assisted laser desorption/ionization time-of-flight mass spectrometry (MALDI-TOF MS) and identified a more diverse mycobiome associated with OSCC, mainly consisting of *Candida* species in addition to *Rhodotorula*, *Saccharomyces*, and *Kloeckera*.

The oral microbiota is dynamic and responsive to environmental and biological changes, so discoverable shifts in its composition and/or function might offer new biomarkers useful for the diagnosis of oral cancers (OCs) and oropharyngeal cancers (OPCs) (Aas et al., 2005). While host biomarkers are subject to individual biological variations (Lim et al., 2017), there are indications that the core oral microbiome is consistently conserved among unrelated subjects (Lim et al., 2017; Shaw et al., 2017). The incorporation of the oral microbiome panel in other tumor biomarkers may therefore help reduce human biological variations, which prevented, so far, the utilization of molecular diagnosis and stratification in OCs and OPCs (Zaura et al., 2014; Lim et al., 2017). Moreover, salivary diagnostics is a rapidly developing field, and combined with biomarker identification and validation, it may provide a platform for the development of a noninvasive, salivary-based tool for the stratification of OSCC patients and for individualized treatments.

This study aimed to investigate the salivary mycobiome in a cohort of OSCC patients and in non-OSCC controls from Sudan and its possible impact on clinical variables, including overall survival (OS). We employed the NGS methodology to explore fungal diversities and communities in saliva and describe the salivary fungal compositional patterns in OSCC patients compared to individuals without OSCC. The fungal genus *Malassezia* was identified as an independent prognostic biomarker for OS of OSCC patients.

## Materials and Methods

### Ethical Considerations

This is a prospective study involving OSCC patients (*n* = 59) and healthy non-cancer controls (*n* = 13) recruited between 2012 and 2015 at Khartoum Dental Teaching Hospital, Sudan. The National Health Research Ethics Committee, Federal Ministry of Health, Sudan, approved the research in Sudan (fmoh/rd/SEC/09). Written informed consent was obtained from both patients and controls. The Regional Ethical Committee in Norway approved the project (REKVest 3.2006.2620 REKVest 3.2006.1341).

### Study Participants

The inclusion criteria were as follows: age older than 18 years, with histologically confirmed primary OSCC, did not receive any previous surgical and chemo- or radiotherapy, and consented to participate in the study. Critically ill patients, patients under medication, and those positive for human immunodeficiency virus (HIV) and hepatitis B surface antigen (HBs Ag) were excluded from the study. Human papilloma virus (HPV)-positive cases were also excluded from the study. Detailed clinical information (age, gender, tobacco habits, and alcohol use) was obtained through interviews. A routine dental examination was performed on participating individuals, which included registration of the periodontal status, plaque, gingival index, community periodontal index (CPI), simplified oral hygiene, fillings and missing teeth, and carious teeth by a team of trained and calibrated dentists specifically for this project. Non-cancer controls were included after informed consent and consecutively recruited from patients attending the outpatient clinic for trauma and benign conditions. The tumor localization, tumor size, TNM stage, comorbid conditions, last date of follow-up, and survival data were obtained from patients’ hospital records. TNM stage was noted according to the guidelines of the American Joint Committee on Cancer, version 7.0. Information on current smoking habits and history of smoking was reported in pack-years (PY) (Masters, 2018), with calculations for consumption of the smokeless tobacco “*toombak*” adjusted according to the average of manually prepared portions in Sudan (Idris et al., 1995).

### Saliva Sample Collection

Unstimulated saliva samples were collected. Briefly, the donor was asked not to eat and not to use oral hygiene products 1 h before saliva collection. At least 2 ml of unstimulated saliva was collected on ice and then kept in a portable liquid nitrogen container until further storage at −80°C at the end of the collection day. The sample collection time did not exceed 20 min.

### Fungal DNA Extraction and Control Sample Setting

The recommendation for standardized DNA extraction for microbiome studies was followed (Leigh Greathouse et al., 2019). A combined enzymatic–mechanical extraction method was chosen and modified, when needed, for fungi (Huseyin et al., 2017; Rosenbaum et al., 2019). Of the saliva, 300 µl was used for DNA extraction. Sputasol^®^ (300 µl, Oxoid Ltd., Basingstoke, UK) was added and incubated, with shaking, at 37°C for 15 min. Following centrifugation, pellets were reconstituted in 250 µl of phosphate-buffered saline (PBS). For enzymatic digestion, an enzyme cocktail of lysostaphin (4,000 U/ml), mutanolysin (25,000 U/ml), and lysozyme (10 mg/ml) was diluted in TE5 buffer (10 mM Tris-HCl and 5 mM EDTA, pH 8.0) (all from Sigma-Aldrich, Saint-Louis, MO, USA). Fifty microliters of the enzyme cocktail was added to each reconstituted pellet and mixed well, then incubated at 37°C with slight shaking at 350 rpm for 1 h. The FastDNA^™^ Kit (MP Biomedicals, Irvine, CA, USA) was used after enzymatic digestion. The samples were centrifuged and the pellets were lysed with 800 µl CLS-Y buffer (FastDNA™ Kit, MP Biomedicals, Irvine, CA, USA). The bead-based protocol for isolation was followed according to the manufacturer’s instructions.

Two biological fungal mock communities (M1 and M2) were included in the study. Both were constituted from environmental fungi: M1 was composed of wood-decomposing polypore fungi (*Mycena galopus*, *Mycena galericulata*, *Mycena leptocephala*, *Mycena epipterygia*, *Serpula lacrymans*, and *Amanita muscaria*), and M2 was constructed from eight fungi isolated from air (*Boeremia exigua* var. *exigua*, *Cladosporium*, *Penicillium chloroleucon*, *Aspergillus fumigatus*, *Discostroma fuscellum*, *Paraphaeosphaeria michotii*, *Mucor hiemalis*, and *Leptosphaerulina chartarum*).

Three single-species positive controls were also prepared from three *Candida* reference strains (*C. albicans* ATCC 10231, *Candida parapsilosis* ATCC 22019, and *Candida glabrata* ATCC MYA-2955).

Serially diluted samples of fungal species isolated from a healthy volunteer and grown on Sabouraud dextrose agar (SDA; Sigma-Aldrich, St. Louis, MO, USA) at 37°C for 48 h were also included as controls. Dilutions (from 1:10 up to 1:10^6^) were done in both artificial saliva (Saliva Orthana^®^, NycoDent, Asker, Norway) and human saliva from a volunteer that did not grow fungi when cultured on SDA. The experimental setup also included three negative controls, two of which were negative extraction controls and the third one just nuclease-free water added before library normalization.

### ITS Amplicon PCR

PCR amplification was performed in a 25-µl reaction volume using 12.5 µl of KAPA HiFi HotStart^®^ ReadyMix PCR Master Mix (Kappa Biosystems, Sigma-Aldrich) and 1 µl of the DNA template, in addition to 0.5 µl of each reverse and forward primers and nuclease-free water. The internal transcribed spacer 2 (ITS2) subregion was targeted for amplification, as recommended (Knot et al., 2009; Nilsson et al., 2019a). ITS2 universal primer 5, 8S ITS2-F GTGAATCATCGARTCTTTGAA, and 28S1 ITS2-R TATGCTTAAGTTCAGCGGGTA (TIB, MOLBIOL, Berlin, Germany) were used to amplify the region of interest. The Veriti Thermal Cycler^®^ (Applied Biosystems, Foster City, CA, USA) was used for amplification. Thermal cycling was done as follows: 3 min at 95°C, initial denaturation followed by 45 cycles of 30 s at 95°C: denaturation, 60 s at 58°C as annealing, 30 s at 72°C for the extension, and a final extension at 72°C for 5 min. The PCR products were examined by electrophoresis in a 1% (*w*/*v*) agarose gel in 1× TAE buffer.

### PCR Clean-up and Library Preparation

Two rounds of clean-up, one after amplicon PCR and the other after index PCR, were performed using a bead-based method (Agencourt AMPure XP, Beckman Coulter, Brea, CA, USA). After the first round, 5 µl from each cleaned up sample was transferred to a 96-well PCR plate for indexing. The indices were arranged according to the manufacturer’s protocols.

### Index PCR and Library Normalization and Denaturation

Nextera XT index primers (Illumina, San Diego, CA, USA) were used for indexing. Index PCR was carried out on the Veriti Thermal Cycler^®^ (Applied Biosystems) with parameters recommended by Illumina (San Diego, CA, USA).

One microliter of a 1:50 dilution of each sample was used for library validation using a Bioanalyzer^®^ DNA 1000 Chip (DNA LabChip^®^ using 2100 Bioanalyzer, Agilent Technologies, Santa Clara, CA, USA). The DNA concentrations of the index PCR products were measured with the Qubit 3.0 Fluorometer^®^ (Invitrogen, Carlsbad, CA, USA), and the DNA concentration was calculated in nanomolars based on the size of the DNA amplicons determined using Bioanalyzer^®^. The normalized library was combined with HT1 and PhiX, as recommended by Illumina.

The MiSeq Reagent Kit v.3 (600 cycles; Illumina, San Diego, CA, USA) was used for library denaturation and MiSeq sample loading. Sequencing was performed on the Illumina MiSeq platform using a 2 × 300-bp paired-end protocol.

### Bioinformatics Processing

Demultiplexed Illumina-generated paired-end sequences were processed using QIIME 2 (version QIIME2-2020.8) (Bolyen et al., 2019). The ITSxpress QIIME 2 plugin (v.1.3) (Rivers et al., 2018) was used to extract the ITS2 region. The sequences were then passed through the DADA2 pipeline (Callahan et al., 2016) for filtration, dereplication, chimera detection, and the merging of paired-end reads to create the so-called amplicon sequence variants (ASVs). The resultant ASVs were included for further analysis. The UNITE database (version 8) (Nilsson et al., 2019b) was trained to create a naive Bayes classifier in order to classify the sequences obtained from the DADA2-generated ASV table. Post-clustering curation using LULU (Frøslev et al., 2017) was performed to avoid diversity overestimation. Unidentified ASVs in the UNITE database were blasted to NCBI and the taxonomy for each was reassigned (considering an *e*-value and similarity or coverage ≥99% of the best hit). Various taxonomic levels were used to classify the sequence data. Species with low abundance (20 reads in less than five samples) were discarded. Three OSCC saliva samples and one non-OSCC control were excluded due to the exclusion criteria for low-abundance samples.

### Statistical Analyses

Differences in the composition of the mycobiome between the OSCC and healthy control groups, and within samples, were tested for significance using relevant statistical tests in MicrobiomeR (Lahti et al., 2017), Phyloseq (McMurdie and Holmes, 2013), and MicrobiomeAnalystR (Dhariwal et al., 2017). Alpha diversity was calculated and plotted in Phyloseq, R version 4.0.3. QIIME2 ANCOM parameters (Bandara et al., 2019) and ALDEx2 (Fernandes et al., 2013) plugins were used for the analysis of the composition of microbiomes. The Kaplan–Meier survival estimator and Cox proportional hazards models (with “enter” method) were used for survival analysis, with OS of 2 years after diagnosis as the end point; all patients who were alive or lost to follow-up at the end of data collection were censored. Survival analysis was performed using Statistical Package for Social Sciences (SPSS), version 25 (IBM, Armonk, NY, USA). For all analyses, *p*-values ≤0.05 were considered to be significant.

## Results

### Cohort Description

The prospective cohort included 59 patients (age range = 25–87 years, mea*n* = 50.6 years, media*n* = 60 years) with histologically proven OSCC and 13 non-OSCC controls (age range = 30–70 years, mea*n* = 46.5 years, media*n* = 45 years). Patients in the OSCC group presented more tobacco consumption (expressed in pack-years for both smoking and smokeless tobacco taken together) than did the controls, although the difference was not statistically significant (*p* = 0.06) (**Table 1**). The average number of decayed teeth (DT) was similar to the general Sudanese population, as previously evaluated (Khalifa et al., 2012), except for the age groups 25–44 and >65 years in our cohort, which showed a higher number of decayed teeth compared to the general Sudanese population. The same was found for missing teeth (MT) (**Table 1**). The mean plaque index of OSCC patients was comparable to that of the control group (*p* = 0.59), while the gingival index (mean ± SD = 1.67 ± 0.56) was significantly higher for the OSCC group (*p* = 0.014) than that for the control group (mean ± SD = 1.19 ± 0.31).

**Table 1:** Demographics, oral health findings, and clinicopathological findings of the cohort.

Cohort demographics									
		*Non-OSCC controls*			*Patients*				
**No. of individuals**		13 (7 males, 6 females)			59 (42 males, 17 females)				
**Age (years), mean**	Males	45.4 (30–60)			60 (25–87)				
	Females	47.7 (39–70)			60.5 (40–80)				
**No. of users**		2 (15%, all males)			33 (56%, all males)				
Pack-years (PY), mean (*p* = 0.06**)		4.5			51.1				
**Oral findings**									
		*Non-OSCC controls*			*OSCC patients*				
**DT (*p* = 0.630)**		2.3			3.9				
**MT (*p* = 0.287)**		6.9			8.8				
Community periodontal index, mean ± SD (CPI: *p* = 0.013*)		1.59 ± 0.67			1.79 ± 0.64				
Gingival index, mean ± SD (*p* = 0.014*)		1.19 ± 0.31			1.67 ± 0.56				
**Missing teeth and decayed teeth in the OSCC cohort** ^a^									
**Age groups (years)**	*DT*				*MT*				
	*Non-OSCC*	*OSCC*		General population*	*Non-OSCC*	*OSCC*		General population*	
**25–34**	0	10		3.3	0	0		1.9	
**35–44**	1.4	4.8		4.1	3.8	3.6		4.2	
**45–54**	3.2	3		4	3.8	6.3		5.5	
**55–64**	5	3.1		3.9	20	8.2		8	
**65–74**	–	4.7		3	32	9.2		11.3	
**75+**	–	3.5		3.3	–	14		11.8	
**Tobacco and alcohol consumption**									
**History**	*Toombak, N (%)*			*Smoking, N (%)*		*Alcohol, N (%)*			
	*Patients*	*Non-OSCC controls*		*Patients*	*Non-OSCC controls*	*Patients*		*Non-OSCC controls*	
**Yes—current user**	5 (8.5)	0 (0)		7 (11.9)	2 (15.4)	1 (1.7)		0 (0)	
**No**	30 (50.8)	11 (84.6)		38	10 (77)	39 (66.1)		11 (86.6)	
**Past user**	22 (37.3)	1 (7.7)		12	0 (0)	13 (22)		1 (1.7)	
**Unknown**	2 (3.4)	1 (7.7)		2 (3.4)	1 (7.7)	6 (10.2)		1 (1.7)	
**OSCC patients: clinical findings**									
***Tumor location***	*N (%)*		*Tumor stage*		*T stage*	*N stage*		*M stage*	
**Buccolabial–sulcus**	32 (54.2)		*N* (%)		*N* (%)		*N* (%)		*N* (%)
				T1	2 (3.4)	N0	4 (6.8)	M0	39 (66.1)
**Tongue**	5 (8.5)	I	0 (0)	T2	11 (18. 6)	N1	19 (32.2)	M1	1 (1.7)
**Retromolar–palatal–alveolar**	15 (25.4)	II	2 (3.4)	T3	15 (25.4)	N2	27 (45.8)	Mx	12 (20.3)
		III	13 (22)	T4	20 (33.9)	N3	1 (1.7)		
		IV	37 (62.7)	Tx	4 (6.8)	Nx	1 (1.7)		
**Missing, *N* (%)**					7 (11.9)

OSCC, oral squamous cell carcinoma; DT, mean number of decayed teeth; MT, mean number of missing teeth.

Significantly different at p < 0.05 (*Kruskal–Wallis and **Mann–Whitney U test).

a Based on Khalifa et al. (2012).

The localization of OSCC lesions was predominantly lower buccal or labial (40.4%); only five cases (6.9%) were localized on the tongue. Of all OSCC patients, 47 (79.6%) presented with locoregional lymph node metastases at the time of diagnosis. Nearly all OSCC patients (96.8%) presented at a late stage (**Table 1**).

### Method Performance

A total of 21,698,808 Illumina-generated demultiplexed fungal ITS raw paired-end sequences were imported into QIIME2. The extracted ITS2 region was merged and temporarily clustered into 6,699,920 amplicon reads. After DADA2 filtration, dereplication, chimera detection, and merging of paired-end reads, a total of 3,514,250 reads were retained for further analysis. The quality-filtered, denoised, chimera-removed sequence reads were clustered into 514 ASVs. Post-clustering curation using LULU (Frøslev et al., 2017) and removal of contaminants using the Decontam algorithm (Davis et al., 2018) retained 340 ASVs. The rarefaction curves curves are presented in **Supplementary Figure 1**.

The positive and negative controls showed the expected reference strains (for positive ones) and negative outputs (negative ones). The M2 mock community showed good distribution, while M1 showed a generally quite good coverage, although taxonomic assignment was obtained correctly only to the class level (*Amanita* and *Mycena* in M1 were identified at the order level, i.e., Agaricomycetes). The composition of our mock communities is reflected in the analysis results, indicating minimal cross-contamination and tag switching. The distribution pattern of the total reads followed the serial dilutions we made (**Supplementary Figure 2**).

Abundance analysis of the serially diluted samples showed a pattern corresponding with the inputs of the diluted samples (**Supplementary Figure 3**).

### *Candida*, *Saccharomyces*, *Malassezia*, *Aspergillus*, and *Cyberlindnera* Were Identified to Be the Most Common Fungi Present in the Salivary Mycobiome

**Figure 1:**
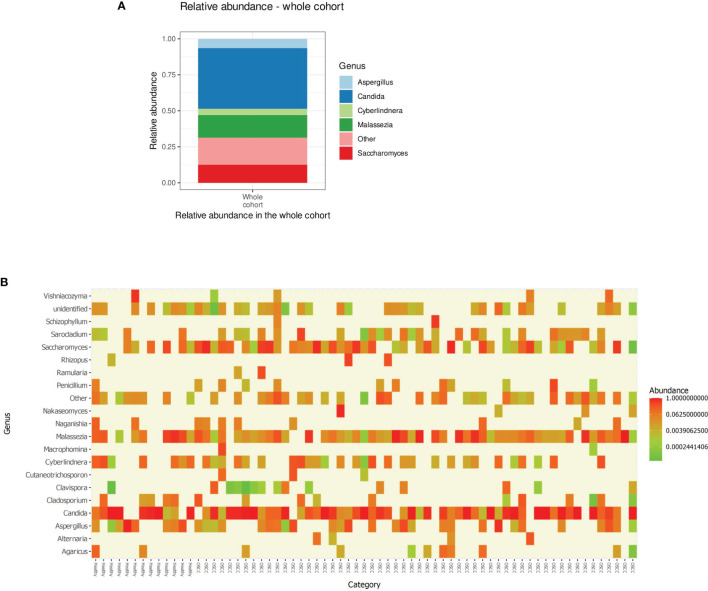
**(A)** Relative abundance of the top five genera in the saliva of the individuals investigated in our cohort. **(B)** Heat map showing the relative abundance of the top 20 genera (*X*-axis sorted non-OSCC controls to the left and OSCC to the right) in each of the investigated sample. OSCC, oral squamous cell carcinoma.

**Figure 2:**
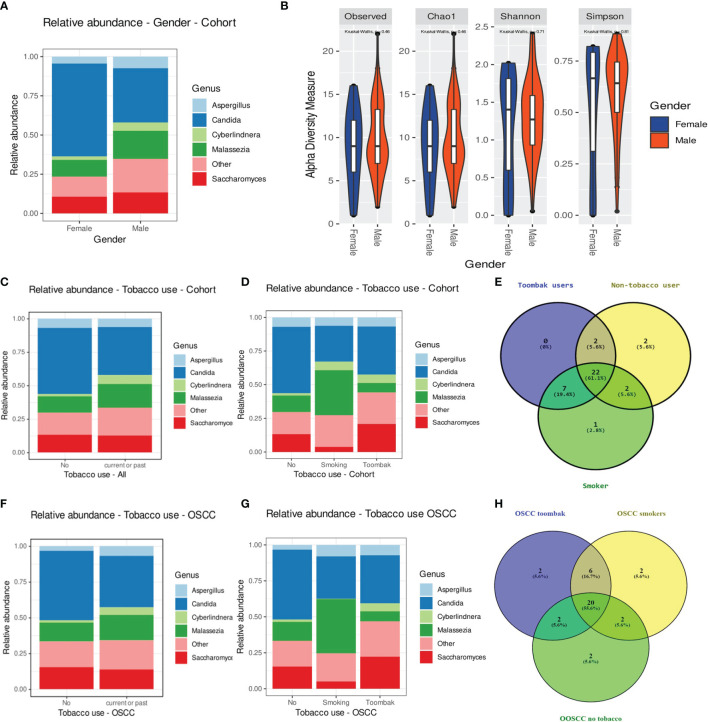
**(A, B)** Relative abundance of the top five genera **(A)** and diversity of the salivary mycobiome **(B)** of the individuals investigated in our cohort grouped by gender. **(C)** Relative abundance of the top five genera in tobacco users *versus* non-smokers. **(D)** Relative abundance of the top five genera in smokers, *toombak* users, and non-smokers. **(E)** Venn diagram showing the distribution of genera in smokers, *toombak* users, and non-smokers. **(F)** Relative abundance of the top five genera in tobacco users *versus* non-smokers in the oral squamous cell carcinoma (OSCC) group. **(G)** Relative abundance of the top five genera in smokers, *toombak* users, and non-smokers in the OSCC group. **(H)** Venn diagram showing the distribution of genera in smokers, *toombak* users, and non-smokers in the OSCC group.

Processed, quality-filtered ASVs were assigned to 36 different fungal genera. Relative abundance analysis showed that the salivary mycobiome was dominated by five genera, namely, *Candida*, *Saccharomyces*, *Malassezia*, *Aspergillus*, and *Cyberlindnera* (**Figure 1A** and **Supplementary Figure 4**). *Agaricus*, *Alternaria*, *Cladosporium*, *Clavispora*, *Naganishia*, *Nakaseomyces*, *Penicillium*, *Rhizopus*, *Vishniacozyma*, and *Sarocladium* were the second most commonly identified fungal genera (**Figure 1B**). *Candida* was found to have a higher relative abundance in the saliva of females than that of males and accounted for more than half of the genera present in females (**Figure 2A**). There was no difference in the diversity of the salivary mycobiome between females and males (**Figure 2B**).

Eight genera were detected exclusively in the saliva of tobacco users (when analyzing together *toombak* dippers and smokers), of which seven were shared by smokers and users of *toombak* (**Figures 2C–E**): *Macrophomina*, *Schizophyllum*, *Cinereomyces*, *Leucosporidium*, *Rhodosporidiobolus*, *Cutaneotrichosporon*, and an unidentified one belonging to the family Ustilaginaceae. *Lodderomyces* was detected only in the saliva of smokers. *Phlebiopsis* and *Filobasidium* were detected only in the saliva of non-tobacco users. No statistically significant differences in the overall oral mycobiome diversity were observed between non-tobacco users and smokers or *toombak* users, even when considering only the OSCC cases (**Figures 2F–H**), although a trend toward somehow restricted diversities in smokers or *toombak* users was observed (**Supplementary Figure 6**).

Individuals aged 55–64 years showed the least relative abundance of *Candida* and the highest abundance of *Aspergillus* in their saliva (**Supplementary Figure 5A**). Individuals with severe gingivitis showed a predominance of species other than the identified top five genera in their saliva (**Supplementary Figure 7A**), along with a gradually reduced diversity, compared to the other two groups. Individuals who needed complex periodontal treatments such as root planing or periodontal surgical procedures (CPI higher than 3) showed a higher relative abundance of *Aspergillus* and a lower relative abundance of *Malassezia* than did those in the other two groups (**Supplementary Figure 7B**). Individuals with intermediate CPI (1.1–3), who needed to undergo plaque control procedures, showed the lowest diversity of fungi compared to other subjects with clinically higher or lower CPIs. Individuals with poor oral hygiene, quantified by the use of a simplified oral hygiene index, showed higher relative abundance of *Candida*, *Aspergillus*, and *Saccharomyces* and a trend toward a lower diversity of fungi (**Supplementary Figure 7C**).

Individuals with the number of decayed teeth higher than that of the mean value for the Sudanese population had a lower relative abundance of *Candida* but a higher relative abundance of *Saccharomyces* than the rest of the participants (**Supplementary Figure 7D**). The opposite was observed for individuals with the number of missing teeth higher than that of the mean value for the Sudanese population (**Supplementary Figure 7E**). The alpha diversity median was also higher, although statistically not significant, for the salivary mycobiome of individuals with more decayed and missing teeth.

### Sixteen Genera Were Identified Exclusively in the Salivary Mycobiome of OSCC Patients

**Figure 3:**
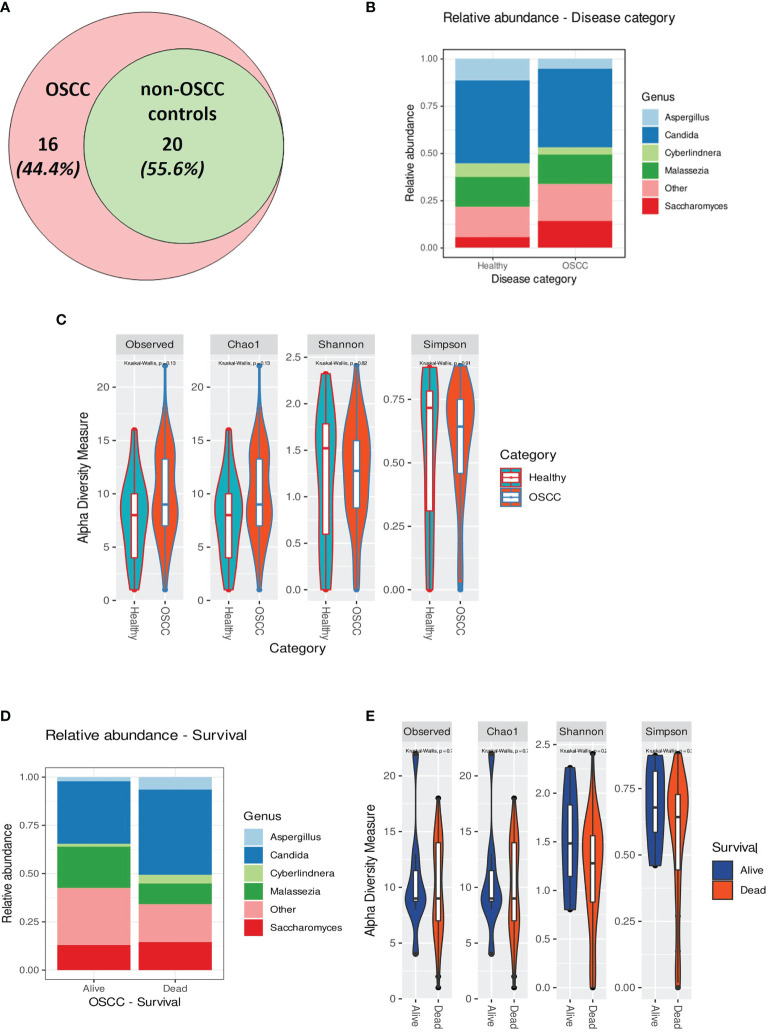
**(A)** Venn diagram showing the number of genera identified in the oral squamous cell carcinoma (OSCC) and non-OSCC groups. Those that were found exclusively in the OSCC group were: *Macrophomina*, *Ramularia*, *Aureobasidium*, *Alternaria*, *Ulocladium*, *Lodderomyces*, *Meyerozyma*, *Schizophyllum*, *Cinereomyces*, *Phlebiopsis*, *Rhodosporidiobolus*, *Rhodotorula glutinis*, *Filobasidium*, *Cutaneotrichosporon*, *unidentified1*, and *unidentified2*. **(B)** Relative abundance in the OSCC and non-OSCC groups showing the top five most predominant genera. **(C)** Alpha rarefaction curve showing the observed features (richness) at different sequencing depths. **(D)** Relative abundance of individuals (alive and dead) in the OSCC groups showing the top five most predominant genera. **(E)** Alpha rarefaction curve showing the observed features (richness) at different sequencing depths for OSCC patients stratified by overall survival (OS).

**Figure 4:**
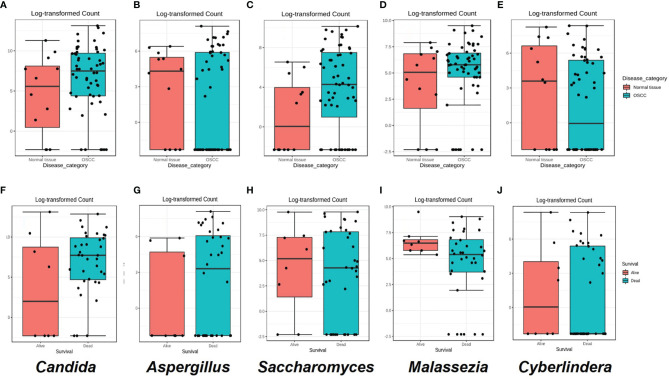
**(A–E)** Box plots for the abundance of the top five genera in the the oral squamous cell carcinoma (OSCC) group *versus* non-OSCC controls. **(F–J)** Box plots for the relative abundance of the top five genera in OSCC patients who were dead or alive at the end of the study period. Classical univariate statistical comparison of the relative abundance showed no statistically significant differences.

The extracted DNA content in the samples from OSCC patients was significantly higher than that in the samples from non-OSCC controls, as evaluated using two different approaches (Qubit^®^ and Bioanalyzer^®^) (*p* < 0.05). Twenty genera were found in the saliva of both the OSCC and non-OSCC groups. Sixteen genera were found exclusively in the saliva of OSCC patients (**Figure 3A**). Univariate statistical comparison of the relative abundance of the top five genera showed no statistically significant differences between the two groups; the same top five most abundant genera were found in both groups (**Figures 3B** and **4**). Alpha diversity analysis, considering richness and evenness, did not show statistically significant differences between OSCC patients and non-OSCC controls (**Figure 3C**). Non-metric multidimensional scaling (NMDS) and analysis of similarities (ANOSIM)/permutational multivariate analysis of variance (PERMANOVA) were applied in order to test for dissimilarities in the mycobiome composition between OSCC patients and non-OSCC controls. There was no shift observed between the study groups (NMDS stress > 0.2); statistical significance was marginal with ANOSIM (*p*_ANOSIM_ = 0.056) and non-significant with PERMANOVA (*p* = 0.265). Differential abundance analysis using ANCOM and ALDex2 did not show any differentially abundant genera when comparing the OSCC group and the non-OSCC control group.

Although not statistically significant, the salivary carriage of *Candida* was higher in the saliva of OSCC cases than that in non-OSCC controls (relative abundance and log-transformed count in each case shown in **Figures 3B** and **4A**, respectively). The *Candida* species identified in the saliva of those in the OSCC group were *C. albicans* (78.8% of all OSCC cases), *Candida tropicalis* (32.1%), *C. parapsilosis* (37.5%), *C. glabrata* (16.1%), *Candida orthopsilosis* (3.6%), and *Candida sake* (9%). *C. orthopsilosis* and *C. sake* were among the fungi identified exclusively in the saliva of OSCC patients.

In the saliva of OSCC cases, *Saccharomyces* also had a higher abundance than that in the saliva of non-OSCC controls (relative abundance and log-transformed count in each case shown in **Figures 3B** and **4C**, respectively). *Saccharomyces cerevisiae* was second to *C. albicans* in the saliva of OSCC cases (76.8% of OSCC cases), while *Malassezia arunalokei* was the second most predominant species in the saliva of non-OSCC controls (66.70% of controls, *n* = 12; 64.3% of OSCC patients, *n* = 56). Additionally, different species of *Malassezia* were identified in the saliva of OSCC patients and non-OSCC controls. *Malassezia globosa* (64.3%), *Malassezia restricta* (16%), *Malassezia dermatis* (5.4%), *Malassezia furfur* (3.5%), and *Malassezia slooffiae* (1.8%) were identified in the saliva of OSCC patients. In the saliva of non-OSCC controls, only *M. restricta* (33.3%) and *M. globosa* (58.3%) were identified.

*Cyberlindnera* had lower abundance in the saliva of OSCC cases than that of non-OSCC controls (relative abundance and log-transformed count in each case shown in **Figures 3B** and **4E**, respectively). *Cyberlindnera jadinii* (synonym: *Pichia jadinii*) was detected in the saliva of 50% of the OSCC patients, while it was present in 58.3% of the non-OSCC controls, showing an inverse relation to *C. albicans* in more than half of the whole group (56% of the whole cohort), although the bivariate correlation was statistically not significant (correlation = −0.257, *p* = 0.1).

### *Malassezia* Was Identified as an Independent Predictor of OS for OSCC Patients

The saliva of OSCC patients with tumors located in labial, buccal, or alveolar areas (*toombak* dipping areas) showed a lower relative abundance of *Candida* but a higher relative abundance of *Cyberlindnera* compared to patients with OSCC located in other sites (**Supplementary Figure 5B**). OSCC patients with locoregional lymph node involvement showed higher relative abundance of *Candida* and *Aspergillus* and a lower relative abundance of *Malassezia* compared to the group with no lymph node involvement (**Supplementary Figure 5C**). The same trend was observed for the OSCC patients who died during the follow-up period compared to those still alive at the end of the study (relative abundance in and log-transformed count in each case shown in **Figures 3D** and **4F–J**, respectively). Alpha diversity analysis revealed that lower diversity index values were more commonly found in OSCC patients with locoregional lymph node involvement and those with poorer survival (**Figure 3E**), although not statistically significant. A trend toward a lower relative abundance of *Saccharomyces* and a higher relative abundance of *Aspergillus* with stage has also been observed (**Supplementary Figure 5D**). Alpha diversity analysis showed no statistically significant differences between stages.

**Figure 5:**
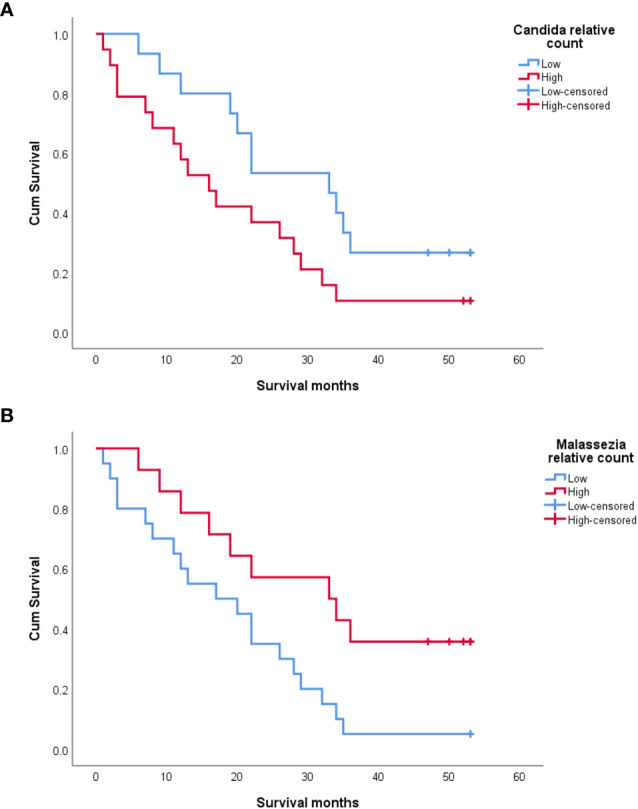
Kaplan–Meier survival curves showing the impact of salivary *Candida* **(A)** and *Malassezia* **(B)** on the overall survival of oral squamous cell carcinoma (OSCC) patients.

Kaplan–Meier analysis revealed that a high relative abundance of *Candida* was associated with poor OS in OSCC patients (Breslow test: *p* = 0.043) (**Figure 5A**). On the contrary, a high relative abundance of *Malassezia* showed association with favorable survival in OSCC patients (Breslow test: *p* = 0.039) (**Figure 5B**). The Cox proportional hazards multiple regression model was applied to adjust the salivary carriage of both *Candida* and *Malassezia* for age (*p* = 0.029) and identified *Malassezia* as an independent predictor of OS (hazard ratio = 0.383, 95% CI = 0.16–0.89, *p* = 0.03).

## Discussion and Conclusion

Although the baseline mycobiome profiles utilizing NGS have been established for some time (Ghannoum et al., 2010; Mukherjee et al., 2014; Chandra et al., 2016), studies on the mycobiome in disease and health are scarce, and the actual contribution of the mycobiota in carcinogenesis has only recently been explored (Perera et al., 2017; Al-Hebshi et al., 2019; Aykut et al., 2019). This study is one of the first characterizing the salivary mycobiome in OSCC and provides significant information despite the fact that it has been run on a relatively smaller number of cases, particularly of non-OSCC controls. Another limitation of this study is that data on antibiotic use were missing. In Sudan, despite instructions, the misuse of antibiotics is a common problem, and the use of antibiotics is known to affect the results of mycobiome analysis (Awad et al., 2007; Oleim et al., 2019).

Most mycobiome studies focused on either the ITS1 or the ITS2 subregion of typically 250–400 bases. Targeting the ITS2 subregion has the additional advantage of including lower length variations and more universal primer sites, resulting in less taxonomic bias than when targeting ITS1 (Nilsson et al., 2019a). In our study, by utilizing 2 × 300-bp sequencing and by merging paired reads, we obtained better taxonomic resolution since the full ITS2 length was covered. We used a relative abundance cutoff of 1%, as used by other studies (Ghannoum et al., 2010; Perera et al., 2017).

The inclusion of negative controls (no saliva sample), positive controls (known species most likely to be found in the samples), and of mock communities was done as a standard for proper assessment and quantification of tag switching, chimera formation, ASV inference stringency, and abundance shifts (Bakker, 2018; Nilsson et al., 2019a). After evaluating the controls, the overall methods used here for DNA extraction, sequencing, and bioinformatics analysis were considered to be sensitive for salivary mycobiome identification, under the conditions and aims of our study.

Although the concept of a healthy core oral mycobiome (Ghannoum et al., 2010) was redefined (Dupuy et al., 2014), with 14 core genera detected in healthy individuals, the overall abundance and diversity of fungal taxa may also be somewhat individualized (Witherden et al., 2017). It is considered that the vast majority of the mycobiome consists of a few genera, with *C. albicans* and *C. parapsilosis* as the major species of the human oral mycobiome (Naglik et al., 2013). The most abundant genera found in our study are in line with these previous baseline findings and with other OSCC-associated salivary mycobiomes reported in previous studies (Ghannoum et al., 2010; Dupuy et al., 2014; Mukherjee et al., 2017; Perera et al., 2017).

Previous studies on the dynamics of the oral bacterial community showed enrichment in both abundance and function with OSCC staging (Yang et al., 2018). We found an enriched but somehow less diverse fungal mycobiome in the most advanced stage group. This might be related to the limited number of cases in the early stages in our cohort. Late tumor stage presentation is typical for OSCC in Sudan (Osman et al., 2010), and as mentioned, this is limiting the conclusions we could draw on the differences between stages in the salivary mycobiome of this cohort.

The salivary microbiome was previously found to be related to dental findings. Gazdeck et al. found a lower bacterial diversity in edentulous patients (Gazdeck et al., 2019). We report here higher diversity median values associated with more missing and decayed teeth. This might indicate relevant fungal–bacterial interactions (Deveau et al., 2018) that need further longitudinal studies for final elucidation. For a long time, our understanding of periodontal disease has been based on its bacterial origin (Hajishengallis and Lamont, 2012). However, the crosstalk between fungi and bacteria seems to result in different outcomes for the host. This relationship ranges from synergism to antagonism for different specific microbial interactions (Krüger et al., 2019). Peters et al. showed *Candida* species to be more represented in subjects with periodontal disease and more missing teeth count (Peters et al., 2017), and, in accordance with this, *C. albicans* was shown to enhance *Porphyromonas gingivalis* invasion *in vitro* (Tamai et al., 2011). We observed the same trend for individuals with higher number of missing teeth.

When it comes to its role in carcinogenesis, in addition to the direct role of *Candida* by producing nitrosamines, it was shown that it also affects the metabolism of procarcinogens and influences other bacteria, which may play a role in carcinogenesis (Hooper et al., 2009). The combinatorial effect of carcinogens and *C. albicans* was shown to promote OC in a murine model (Dwivedi et al., 2009). *C. albicans* was also shown to enhance the invasion of OSCC cells by producing specific proteinases capable of degrading the basement membrane and the extracellular matrix (Bakri et al., 2010). The inflammatory response to *C. albicans* is mediated by NF-κB (Müller et al., 2007), which is frequently involved in carcinogenesis where cancer-related inflammation is a feature (Mantovani et al., 2008). Taking this into consideration, our finding of the association between *Candida* and poor prognosis might rely on a biological explanation.

On the other hand, we found the salivary carriage of *Malassezia* as an independent predictor of better prognosis. *Malassezia* has a unique pattern of interaction with pattern recognition receptors compared to *C. albicans* (Goyal et al., 2018). Additionally, *Malassezia* has large intraspecies diversity. The exact composition of different *Malassezia* species at a time point may contribute to different outcomes in the interaction between the fungus and the host (Sparber and LeibundGut-Landmann, 2017). *Malassezia* might have been overrepresented in our study, although it was described as part of the redefined core oral mycobiome in humans (Dupuy et al., 2014). Since *Malassezia* is a normal skin commensal with population densities peaking between 20 and 45 years (Ashbee, 2007), the sample collection method we used might have included some contamination from the lips, in addition to the age-related differences in *Malassezia* enrichment. However, the interest in discovering microbiome profiles associated with survival is growing (Plantinga et al., 2017; Koh et al., 2018). Different methods are used to associate the microbiota at the community level and censored survival time, such as MiRKAT-S (Plantinga et al., 2017) and its follower OMiSA (Koh et al., 2018). We chose to test the categorization of microbial proportions into high and low and run conventional survival analysis. This seems an attractive way to incorporate microbiome signatures in clinically applicable diagnostic tools.

Of interest is that we did not identify *Hannaella* and *Gibberella*, which were found enriched in a cohort of OSCC patients in a recent study and considered to be contaminants (Perera et al., 2017). This might be an indication of the effects of dietary habits, among others, and population-related differences in the mycobiome profiles. These two species are known plant fungi. Nevertheless, the contribution of environment-related fungi to the carcinogenic process cannot be ignored.

The sample type, the method of collection, and, very importantly, the methods for DNA extraction and bioinformatics processing could explain the observed differences between species reported in different studies (Brooks et al., 2015). Curation of the databases used for taxonomical assignment could also affect the findings (Seed, 2015), in addition to the more classical factors such as ethnic differences and diet (Deschasaux et al., 2018). Ethnic differences could be related to different single nucleotide polymorphisms associated with susceptibility to fungi (Romani, 2011). The role of genetic host susceptibility should not be ignored when considering the diversity changes or the associations of the mycobiota with diseases. In any case, a further, more thorough investigation of mycobiome meta-transcriptomes and metaproteomes is needed to answer such questions related to the epidemiological patterns of mycobiomes (Huttenhower et al., 2012).

Also worth mentioning is the fact that the cohort analyzed in this study included patients consuming a special type of smokeless tobacco, the “*toombak*” (the local form of smokeless tobacco used in Sudan). Regarding our findings on tobacco consumption (both smoking and the smokeless tobacco *toombak*), it is worth noting that there may be bias related to self-reporting. However, the predominant site for tumor localization in our OSCC cohort was lower buccal or labial and sulcular, consistent with a *toombak*-related OSCC etiology, and this also correlated with the self-reported habit of packing *toombak* in the mouth in our cohort. Self-reporting of alcohol consumption should be considered with caution as well since it is illegal in Sudan and may carry a social stigma (Gadelkarim Ahmed and Ahmed, 2013). Previous studies have shown that tobacco exposure was associated with a shift of the oral bacteriome at the population level (Beghini et al., 2019). Our study showed the same trend for the oral mycobiome. Some of the genera were identified exclusively in tobacco users, including *toombak* users, since many consume *toombak* besides smoking, and some of the genera, such as *Schizophyllum*, are known plant pathogens; thus, they may be related to the processed tobacco product.

In conclusion, the present study reveals that *Candida*, *Malassezia*, *Saccharomyces*, *Aspergillus*, and *Cyberlindnera* are the most relatively abundant fungal genera in the salivary microbiome of this cohort of Sudanese individuals. *Candida* and *Malassezia* were shown to have an impact on the survival of OSCC patients: a higher salivary carriage of the genus *Candida* was found to be associated with poor prognosis, while *Malassezia* was enriched in patients with favorable prognosis, although only the salivary carriage of *Malassezia* emerged as an independent prognostic biomarker for the survival of OSCC patients. This can serve as groundwork for performing mycobiome-based biomarker studies in larger cohorts of OSCC patients.

## Data Availability Statement

The raw data represented in this study has been deposited and is publicly available from SRA (PRJNA722859); NCBI PRJNA722859.

## Ethics Statement

The National Health Research Ethics Committee, Federal Ministry of Health, Sudan, approved the research in Sudan (fmoh/rd/SEC/09). Also, the Regional Ethical Committee in Norway approved this project (REKVest 3.2006.2620 REKVest 3.2006.1341). The patients/participants provided their written informed consent to participate in this study.

## Author Contributions

NM and DC conceptualized the study. NM, JL, IA, EM, JF, RJ-L, LM, SM, TO, and EN helped with the methodology. NM, EM, JF, RJ-L, LM, SM, TO, and EN did the formal analysis. NM, JL, IA, ME, TO, EN, AS, AJ, and DC contributed to the investigation. NM prepared the original draft. All co-authors reviewed and edited the manuscript. NM, EM, JF, RJ-L, SM, TO, and DC contributed to visualization. AJ, TO, EN, AS, and DC supervised the study. EN, AS, AJ, and DC administered the project. AJ and DC helped with funding acquisition. All authors contributed to the article and approved the submitted version.

## Funding

This work was supported by the Research Council of Norway through its Centers of Excellence funding scheme (grant no. 22325), Helse Vest (grant no. 911902/2013 and 912260/2019), and the Norwegian Centre for International Cooperation in Education (project no. NORPART-2018/10277).

## Conflict of Interest

The authors declare that the research was conducted in the absence of any commercial or financial relationships that could be construed as a potential conflict of interest.

## Publisher’s Note

All claims expressed in this article are solely those of the authors and do not necessarily represent those of their affiliated organizations, or those of the publisher, the editors and the reviewers. Any product that may be evaluated in this article, or claim that may be made by its manufacturer, is not guaranteed or endorsed by the publisher.

## Supplementary Material

Supplementary Figure 1 Sequencing depth curves for OSCC and non-OSCC controls.

Supplementary Figure 2 Pie charts depicting expected and detected distribution of mock communities. Stars refers to identical genera detected.

Supplementary Figure 3 Bar-graph representing the number of reads according to the serial dilution of samples. Y axis represents log scale of proportions. AS - sample diluted in Artificial Saliva, HS - sample diluted in Human Saliva that did not grow fungi when cultured on Sabraud’s Dextrose agar medium.

Supplementary Figure 4 Bar plots showing the relative abundance in saliva of OSCC cases and non-OSCC controls. Five most dominant genera were shown.

Supplementary Figure 5
**(A)** Distribution and differences in abundance of 5 topmost salivary fungi according to age. **(B)** Distribution and differences in abundance of 5 topmost salivary fungi according to tumour localization. **(C)** Distribution and differences in abundance of 5 topmost salivary fungi according to lymph node metastasis status. **(D)** Distribution and differences in abundance of 5 topmost salivary fungi according to tumour stage.

Supplementary Figure 6 Diversity of the overall oral mycobiome of the individuals of our cohort grouped by tobacco use (no tobacco users, smokers and *Toombak* users).

Supplementary Figure 7
**(A)** Distribution and differences in abundance of 5 topmost salivary fungi according to Gingival Index. **(B)** Distribution and differences in abundance of 5 topmost salivary fungi according to Community Periodontal Index. **(C)** Distribution and differences in abundance of 5 topmost salivary fungi according to Oral Hygiene Index (Simplified). **(D)** Distribution and differences in abundance of 5 topmost salivary fungi according to mean number of decayed teeth. **(E)** Distribution and differences in abundance of 5 topmost salivary fungi according to mean number of missing teeth. **(F)** Distribution and differences in abundance of 5 topmost salivary fungi according to diabetes status.
