# Supplementary figures and images for: Analysis of Salivary Mycobiome in a Cohort of Oral Squamous Cell Carcinoma Patients From Sudan Identifies Higher Salivary Carriage of Malassezia as an Independent and Favorable Predictor of Overall Survival
Source: Front Cell Infect Microbiol. 2021 Oct 12;11:673465. doi: 10.3389/fcimb.2021.673465 (PMC8547610; doi:10.3389/fcimb.2021.673465)

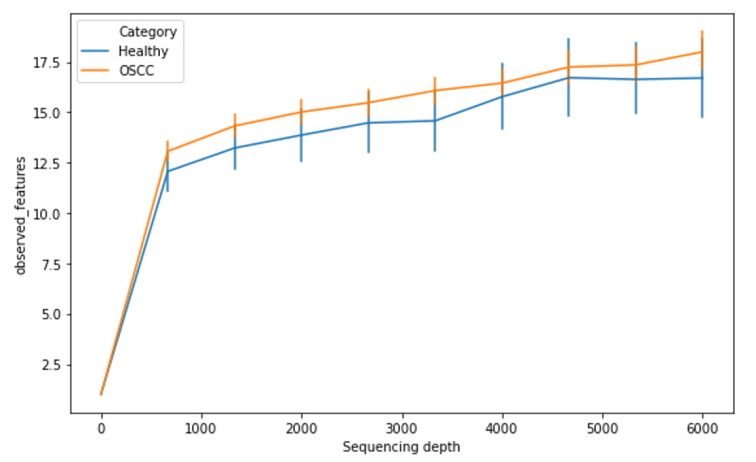

Supplement: Supplementary Figure 1 — Sequencing depth curves for OSCC and non-OSCC controls. [file Image_1.jpeg]

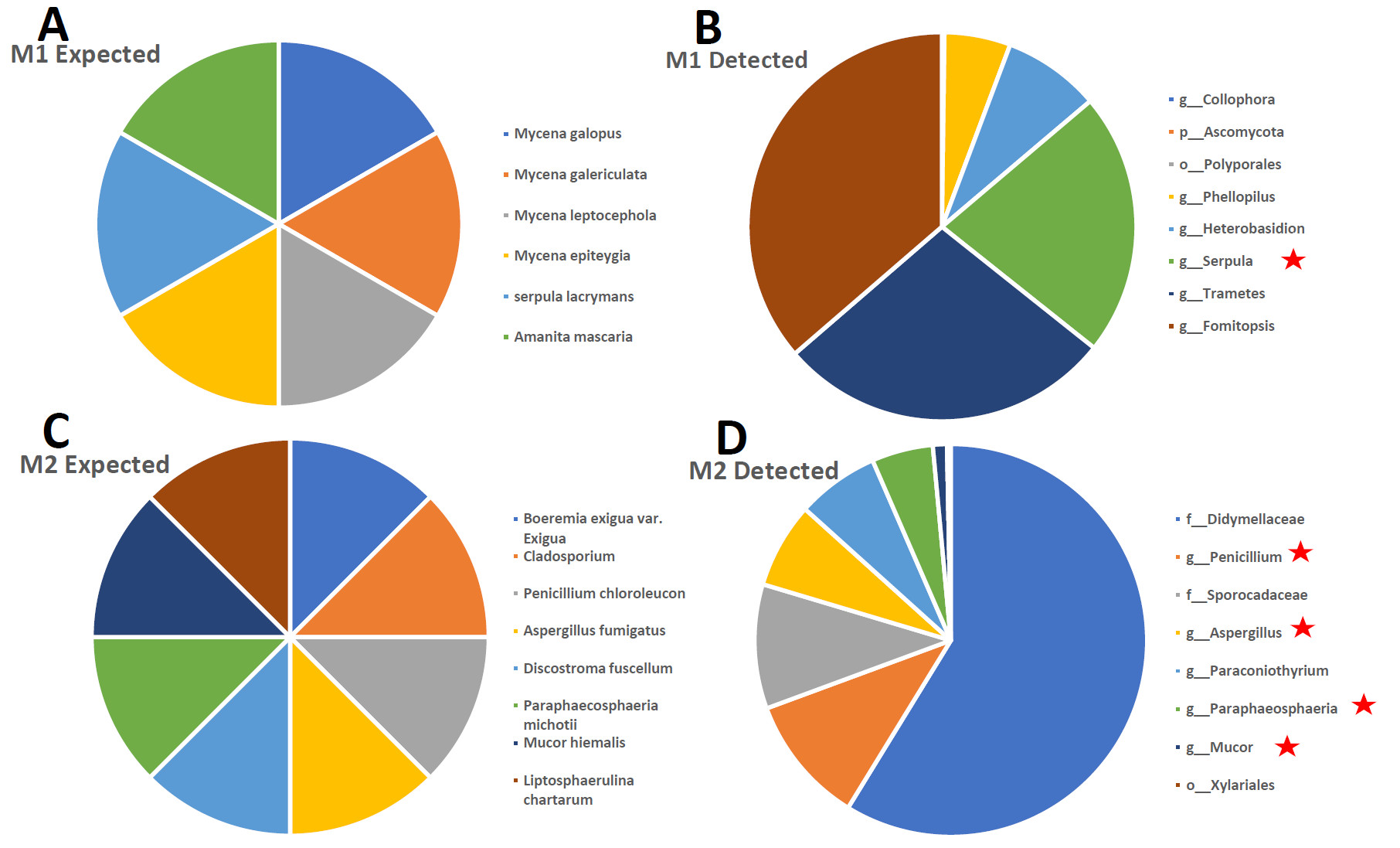

Supplement: Supplementary Figure 2 — Pie charts depicting expected and detected distribution of mock communities. Stars refers to identical genera detected. [file Image_2.jpg]

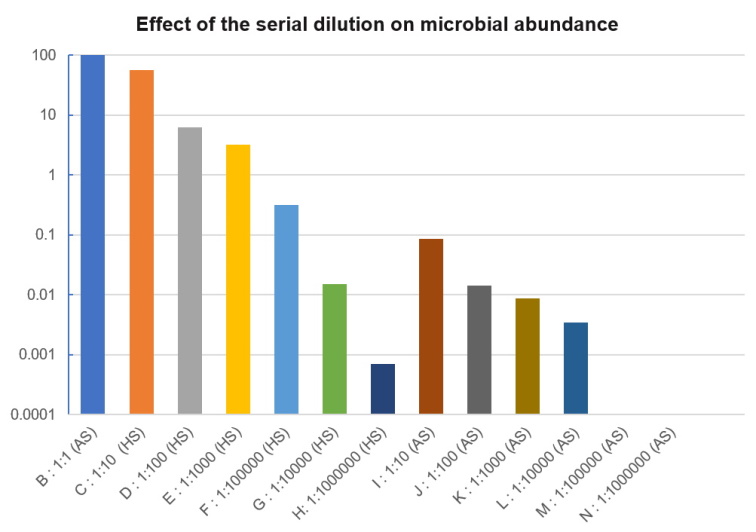

Supplement: Supplementary Figure 3 — Bar-graph representing the number of reads according to the serial dilution of samples. Y axis represents log scale of proportions. AS - sample diluted in Artificial Saliva, HS - sample diluted in Human Saliva that did not grow fungi when cultured on Sabraud’s Dextrose agar medium. [file Image_3.jpeg]

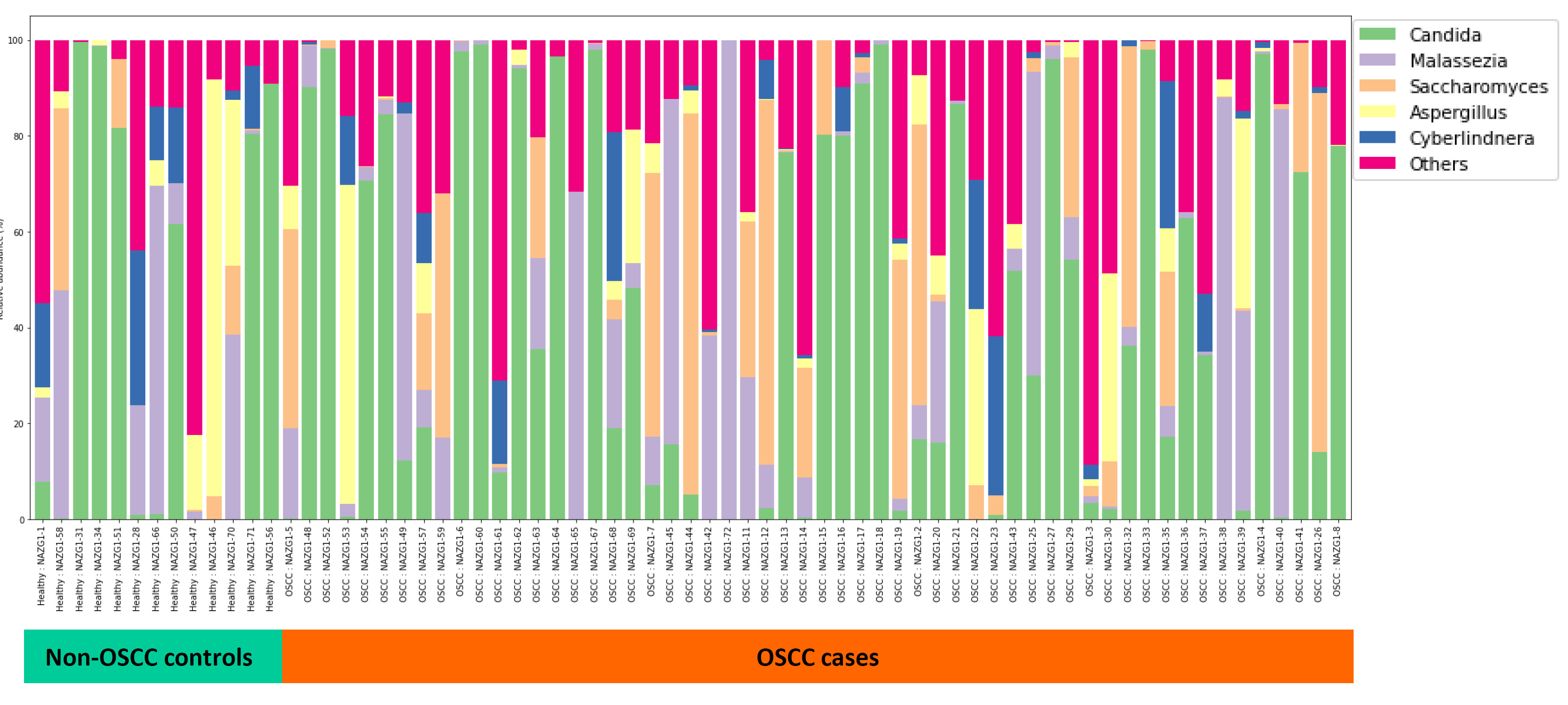

Supplement: Supplementary Figure 4 — Bar plots showing the relative abundance in saliva of OSCC cases and non-OSCC controls. Five most dominant genera were shown. [file Image_4.jpg]

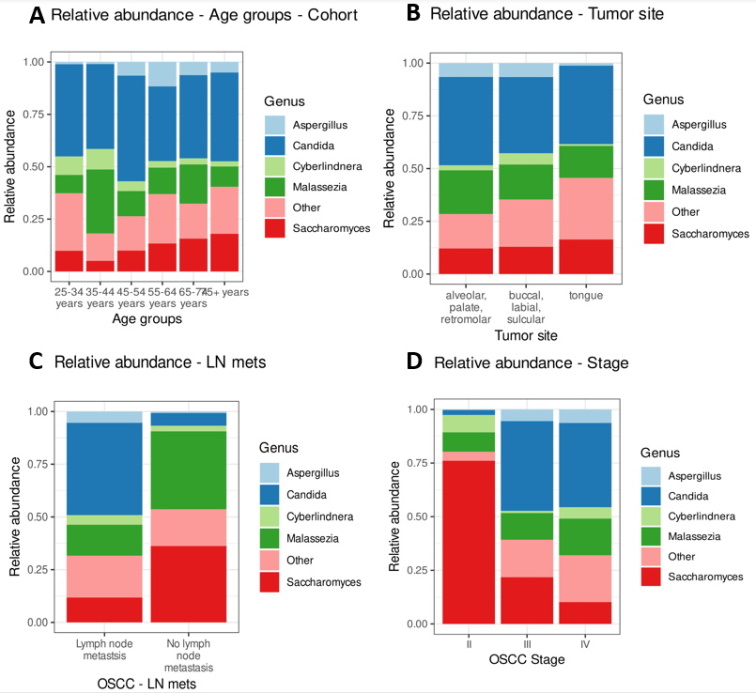

Supplement: Supplementary Figure 5 — (A) Distribution and differences in abundance of 5 topmost salivary fungi according to age. (B) Distribution and differences in abundance of 5 topmost salivary fungi according to tumour localization. (C) Distribution and differences in abundance of 5 topmost salivary fungi according to lymph node metastasis status. (D) Distribution and differences in abundance of 5 topmost salivary fungi according to tumour stage. [file Image_5.jpeg]

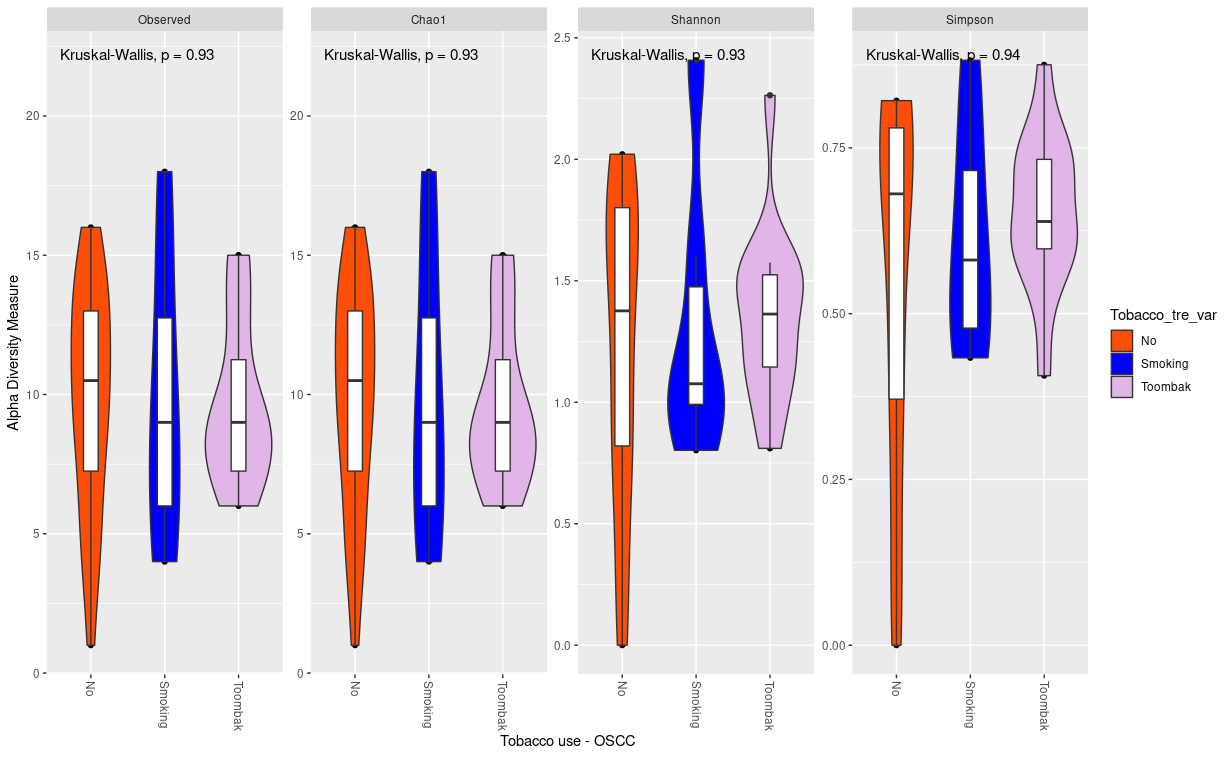

Supplement: Supplementary Figure 6 — Diversity of the overall oral mycobiome of the individuals of our cohort grouped by tobacco use (no tobacco users, smokers and Toombak users). [file Image_6.png]

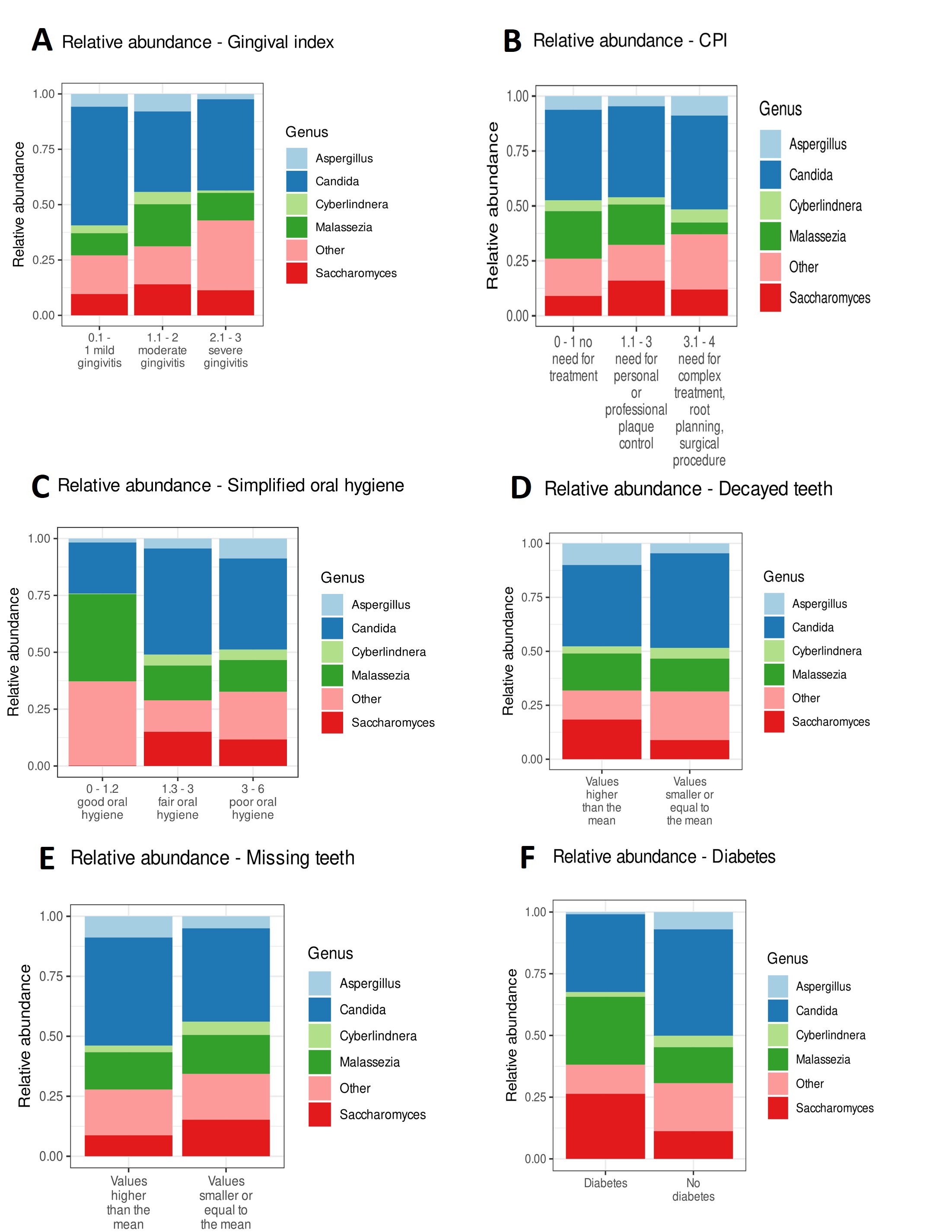

Supplement: Supplementary Figure 7 — (A) Distribution and differences in abundance of 5 topmost salivary fungi according to Gingival Index. (B) Distribution and differences in abundance of 5 topmost salivary fungi according to Community Periodontal Index. (C) Distribution and differences in abundance of 5 topmost salivary fungi according to Oral Hygiene Index (Simplified). (D) Distribution and differences in abundance of 5 topmost salivary fungi according to mean number of decayed teeth. (E) Distribution and differences in abundance of 5 topmost salivary fungi according to mean number of missing teeth. (F) Distribution and differences in abundance of 5 topmost salivary fungi according to diabetes status. [file Image_7.jpeg]
